# Age-Related Changes in Lipidome of Rat Frontal Cortex and Cerebellum Are Partially Reversed by Methionine Restriction Applied in Old Age

**DOI:** 10.3390/ijms222212517

**Published:** 2021-11-20

**Authors:** Mariona Jové, Rosanna Cabré, Natàlia Mota-Martorell, Meritxell Martin-Garí, Èlia Obis, Paula Ramos, Iván Canales, José Daniel Galo-Licona, Joaquim Sol, Lara Nogueras, Pascual Torres, Manuel Portero-Otín, Victòria Ayala, Isidro Ferrer, Reinald Pamplona

**Affiliations:** 1 Department of Experimental Medicine, University of Lleida-Lleida Biomedical Research Institute (UdL-IRBLleida), E-25198 Lleida, Spain; mariona.jove@udl.cat (M.J.); rosannacabre@gmail.com (R.C.); nataliamotamartorell@gmail.com (N.M.-M.); meritxell.martin@udl.cat (M.M.-G.); elia.obis@udl.cat (È.O.); paula060497@gmail.com (P.R.); ivancanalesalandi97@gmail.com (I.C.); jgalolic25@gmail.com (J.D.G.-L.); jsol.lleida.ics@gencat.cat (J.S.); lara.noguerasp@gmail.com (L.N.); pascual.tc92@gmail.com (P.T.); manuel.portero@udl.cat (M.P.-O.); victoria.ayala@udl.cat (V.A.); 2 Institut Català de la Salut, Atenció Primària, E-25007 Lleida, Spain; 3 Research Support Unit Lleida, Fundació Institut Universitari per a la Recerca a l’Atenció Primària de Salut Jordi Gol i Gurina (IDIAPJGol), E-25007 Lleida, Spain; 4 Department of Pathology and Experimental Therapeutics, Institute of Biomedical Research of Bellvitge (IDIBELL), University of Barcelona, E-08907 L’Hospitalet de Llobregat, Spain; 8082ifa@gmail.com; 5 Center for Biomedical Research on Neurodegenerative Diseases (CIBERNED), Institute of Health Carlos III, E-28220 Madrid, Spain

**Keywords:** aging, glycerolipids, glycerophospholipids, (lip)oxidation-derived protein damage markers, mass spectrometry, methionine restriction, sphingolipids, sterol lipids

## Abstract

Lipids are closely associated with brain structure and function. However, the potential changes in the lipidome induced by aging remain to be elucidated. In this study, we used chromatographic techniques and a mass spectrometry-based approach to evaluate age-associated changes in the lipidome of the frontal cortex and cerebellum obtained from adult male Wistar rats (8 months), aged male Wistar rats (26 months), and aged male Wistar rats submitted to a methionine restriction diet (MetR)—as an anti-aging intervention—for 8 weeks. The outcomes revealed that only small changes (about 10%) were observed in the lipidome profile in the cerebellum and frontal cortex during aging, and these changes differed, in some cases, between regions. Furthermore, a MetR diet partially reversed the effects of the aging process. Remarkably, the most affected lipid classes were ether-triacylglycerols, diacylglycerols, phosphatidylethanolamine N-methylated, plasmalogens, ceramides, and cholesterol esters. When the fatty acid profile was analyzed, we observed that the frontal cortex is highly preserved during aging and maintained under MetR, whereas in the cerebellum minor changes (increased monounsaturated and decreased polyunsaturated contents) were observed and not reversed by MetR. We conclude that the rat cerebellum and frontal cortex have efficient mechanisms to preserve the lipid profile of their cell membranes throughout their adult lifespan in order to maintain brain structure and function. A part of the small changes that take place during aging can be reversed with a MetR diet applied in old age.

## 1. Introduction

The abundance and diversity of lipid classes and molecular species present in the nervous system of animal species is evidence of the relevance of this family of compounds for the structure and function of neural cells [1,2]. Structural components of cell membranes, signaling role, energy metabolism, and vulnerability to oxidative damage are properties ascribed to brain lipids [2,3]. The maintenance of this molecular complexity is so demanding that one quarter of total brain energy expenditure must be invested in the cellular activity involved in the homeostatic regulation of the brain lipid metabolism [4] including de novo lipid biosynthesis, remodeling, turnover, and synthesis of bioactive lipids, as well as the continuous adjustment of spatial and temporal lipid organization of cell membranes [2,5,6]. The result is that the nervous system has a lipid profile that diverges significantly from that of other non-neural tissues [7].

There is evidence linking changes in the lipidome with aging and longevity (reviewed in [8,9]). The physiological aging process and several neurodegenerative diseases are associated with changes in the brain lipid profile, which seem to be region-specific [3,10,11,12]. Lipid binding to proteins involved in age-related diseases also plays an important, although not completely understood, role in aging [13]. Yet, evidence about lipid profile changes in the rat brain during aging is limited. Previous studies on rat brains suggested age-related changes of individual lipid classes such as sterols [14], glycerophospholipids [15,16,17], and gangliosides [17,18] in the whole brain [18] or specific brain regions such as the amygdala, cerebral cortex, cerebellum, frontal cortex, hippocampus, and hypothalamus [14,15,16,17,19]. However, most of these studies involved a partial analysis of the lipidome, focusing on specific lipid categories or classes.

The aging process can be delayed by dietary interventions [20,21]. Diverse dietary restrictions extend longevity in animal species, inducing a metabolic reprogramming which is phenotypically expressed with a reduction in oxidative stress, and adaptation and optimization of lipid profiles at the tissue level [8,20,21]. Notably, these anti-aging effects can be reproduced with a dietary methionine restriction (MetR) [20,21]. MetR reduces oxidative stress and oxidation-derived molecular damage, and it modifies the lipid profile in the whole brains of rats and mice [22,23,24,25]. However, all the studies published to date applying MetR were performed using young animal specimens, and the effect of MetR in aged animals is currently unknown. Furthermore, evaluation of the effect of a short-term MetR intervention at old age in animals that have been feeding ad libitum throughout their lifespan is crucial for future anti-aging interventions.

In this scenario, the main goal of the present study is to reveal if a short period of MetR applied in old age is advantageous for animals that have been feeding ad libitum during their lifespan. In trying to overcome those boundaries, we have studied the effect of short-term (7 weeks) MetR in 26-month-old Wistar rats. We have designed a study including three experimental groups: Adult (8 months old, control diet), Aged (26 months old, control diet), and Aged+MetR (26 months old, 80% MetR diet). In order to evaluate the effect of MetR in the cerebellum and frontal context lipidome, gas chromatography (GC), gas chromatography–mass spectrometry (GC/MS), and liquid chromatography–mass spectrometry (LC-MS)-based untargeted lipidomic approaches were used to analyze the total lipid fatty acid composition, the concentration of specific (lip)oxidation-derived protein damage markers, and the lipidomic profiles, respectively. In this way, we studied lipid differences in two distinct regions of the rat brain that are involved in motor and cognitive functions, but showing different vulnerability to the aging process [26,27]. Despite the presence of interspecies differences, it seems that there is an important component in the frontal cortex and cerebellum function across the mammalian class [28]. Recognizing this fact allows us to establish a certain degree of analogy between potential changes in these brain regions in rodents and what may happen in humans.

## 2. Results

### 2.1. Effect of Aging and Methionine Restriction in the Cerebellum Lipidome

**Figure 1:**
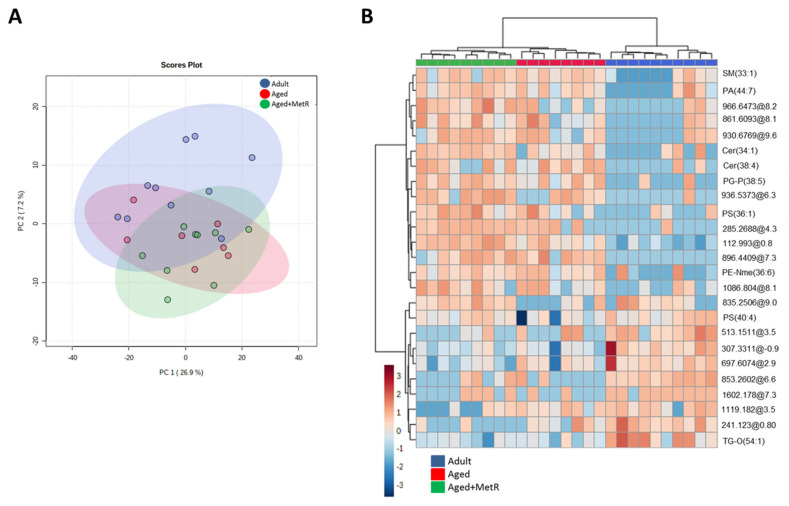
Multivariate analysis of the differential lipid species detected in the cerebellum during aging and methionine restriction applied in old age. (**A**) Two-dimensional principal component analysis (PCA) for the different analyzed groups: adult, aged, and aged+MetR. (**B**) Heatmap of hierarchical clustering using the 25 lipid species with the lowest *p*-values. Each colored cell on the map corresponds to a relative concentration value, with samples in columns and compounds in rows. n (Adult) = 7, n (Aged) = 9, n (Aged+MetR) = 10.

In the cerebellum, our global lipidome analysis detected a total of 13,949 molecular features from both ionization modes (positive and negative) once baseline correction, peak picking, and peak alignment were applied on acquired data. After quality control assessment, filtering, and correcting the signal, 665 features remained, which were used for statistical analysis. Principal component analysis (PCA) showed that the three first components (PC1, PC2, and PC3) explained 42.4% of the variability of the samples. This analysis showed that although the changes in the global lipidome are minor, both aged groups (Aged and Aged+MetR) were more homogenous and similar to each other than to the adult group, indicating that aging, more than dietary intervention, is determinant in the definition of the lipidome in the cerebellum (Figure 1A). Multivariate statistics applied only to adult and aged groups revealed that there was an almost perfect clusterization according to their age group (Supplementary Figure S1A).

Partial least-squares discriminant analysis (PLS-DA) was able to clearly separate the three groups (data not shown), but permutation tests (1000 repeats) yielded a not significant *p* value (*p* = 0.33), indicating that it is not an optimal model. Hierarchical clustering using all lipid species detected showed there was no specific trend when the whole lipidome was taken into account (Supplementary Figure S2A). However, when this analysis was performed using the 25 lipid species with the lower *p* value (Figure 1B), it confirmed that the aging process affected the cerebellum lipidome more than the MetR diet. The heatmap of metabolite abundances showed that the adult group had a specific profile compared with the other two groups.

Lipidomics analysis demonstrated the existence of specific changes in the lipidome of the cerebellum during aging and diet applied in old age. Thus, when we searched for specific aging and diet biomarkers, we found 60 lipid species to be statistically different between groups (*p* < 0.05) (Table 1, Supplementary Materials, http://metrlipidombrain.pythonanywhere.com/, accessed on 16 November 2021). Among all the significant molecules, 24 were annotated, and 32 were not identified. Among identified lipid species, we described 6 glycerolipids (GL), 10 glycerophospholipids (GP), 5 sphingolipids (SP), and 3 sterol lipids (SL). According to the differences between groups, we assigned a biological meaning for the differential lipid species consisting of three concepts: healthy aging, aging, and diet. For healthy aging, the lipid species that followed the post hoc were considered: Aged ≠ Aged+MetR and Adult. For a lipid species to be considered as biomarker of aging, the post hocs were Adult ≠ Aged and Aged+MetR. Finally, as biomarkers of the effect of MetR, we considered Aged+MetR ≠ Aged and Adult. Following this rule, we found 27 biomarkers of aging (2 identified as GL, 5 as GP, 4 as SP, and 1 as SL), 11 biomarkers of MetR diet (1 identified as GL, 2 as GP, and 1 as SL) (Supplementary Figure S2), and 4 biomarkers of healthy aging (1 identified as GL, 1 as GP, and 1 as SL).

After analyzing changes in the global lipidome, we focused on fatty acid composition of total lipids. This analysis in the cerebellum showed a significant increase of 14:0 (11%), 20:0 (18%), and 20:1n-9 (10%) in the aged rat. In contrast, the abundance of PUFA such as 20:2n-6, 20:4n-6, and 22:4n-6 was lower in aged rats compared to adult rats, by 12%, 6%, and 15%, respectively (Table 2). As result of these minor but significant changes, we observed increased content of monounsaturated fatty acids (MUFA) (5%), and decreased content of PUFA (5%), particularly PUFAn-6 (7%), in the aged group (Table 3). These compositional changes determined a lower double bond index (DBI) and peroxidizability index (PI) (Table 3), suggesting a lipid membrane more resistant to oxidative damage. In this line, no changes, or even a significant decrease, were observed in the different oxidation-derived protein damage markers analyzed in the aged group compared to the adult group (Table 4). The estimation of enzymatic activities related to the modification of the degree of unsaturation (desaturase activity) and chain length (elongase) of fatty acid showed specific decreased estimated elongase activities, with desaturase activities sustained with aging (Table 3). All these changes with aging were maintained in cerebellum despite the introduction of methionine restriction at old age (Table 2, Table 3 and Table 4).

### 2.2. Effect of Aging and Methionine Restriction in the Frontal Cortex Lipidome

**Figure 2:**
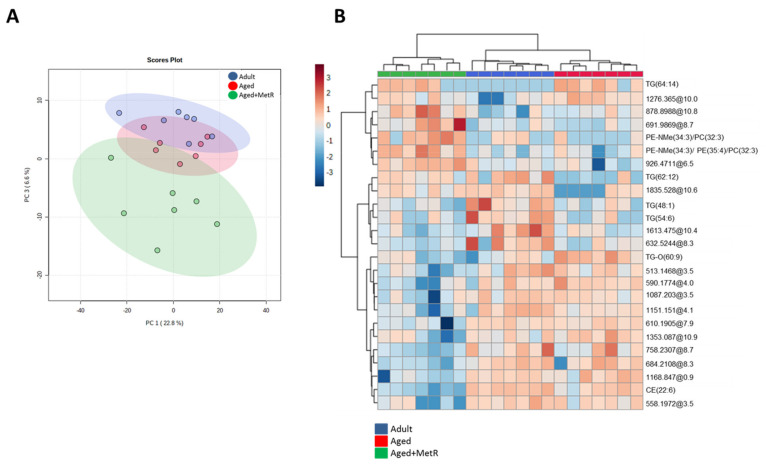
Multivariate analysis of the differential lipid species detected in the frontal cortex during aging and methionine restriction applied in old age. (**A**) Two-dimensional principal component analysis (PCA) for the different analyzed groups: adult, aged, and aged+MetR. (**B**) Heatmap of hierarchical clustering using the 25 lipid species with the lowest *p*-values. Each colored cell on the map corresponds to a relative concentration value, with samples in columns and compounds in rows. N (Adult) = 7, n (Aged) = 7, n (Aged+MetR) = 7.

In the frontal cortex, our lipidomics analysis detected a total of 11,029 molecular features from both ionization modes (positive and negative), once baseline correction, peak picking, and peak alignment were applied on acquired data. After quality control assessment, filtering, and correcting the signal, 763 features remained, which were used for statistical analysis. Using the whole lipidome, principal component analysis (PCA) showed that the three first components (PC1, PC2, and PC3) explained 36.8% of the variability of the samples. This analysis showed that the adult group lipidome differed with respect to the aged groups, and that the MetR group was also clearly differentiated, suggesting that aging and diet may be determinants in the definition of the lipodome in the frontal cortex (Figure 2A). Multivariate statistics applied only to adult and aged groups revealed that the changes in the central cortex lipidome were minor during aging (Supplementary Figure S1B).

Partial least-squares discriminant analysis (PLS-DA) was able to clearly separate the three groups (data not shown), but permutation tests (1000 repeats) yielded a not significant *p* value (*p* = 0.17), indicating that it was not an optimal model. Hierarchical clustering using all lipid species detected showed no specific trend when the whole lipidome was analyzed (Supplementary Figure S2B). However, a heatmap of relative intensity changes for the 25 lipid species with the lower *p* value was then composed to visualize possible clustering (Figure 2B), and a pattern emerged suggesting that although all three groups grouped perfectly, diet was the most important factor determining the frontal cortex lipidome. The heatmap of metabolite abundances showed that the Adult+MetR group had a specific profile compared with the other two groups.

Lipidomics analysis demonstrated the existence of specific changes in the lipidome of the frontal cortex during aging and MetR applied in old age. Thus, when we searched for specific aging and diet biomarkers, we found 81 lipid species to be statistically different between groups (Table 5, Supplementary Materials, http://metrlipidombrain.pythonanywhere.com/, accessed on 16 November 2021). Among all the significant molecules, 34 were annotated, and 41 were not identified. Among identified lipid species, we described 1 FA, 11 GL, 14 GP, 5 SP, 1 prenol lipid (PL), and 2 SL. According to the differences between groups, we assigned a biological meaning for the differential lipid species consisting of three concepts: healthy aging, aging, and diet. Healthy aging was considered the lipid species that followed the post hoc: Aged ≠ Aged+MetR and Adult. To consider a lipid species as biomarker of aging, the post hocs were: Adult ≠ Aged and Aged+MetR. Finally, biomarkers of the effect of MetR were considered: Aged+MetR ≠ Aged and Adult. Following this rule, we found 25 biomarkers of aging (1 identified as FA, 4 as GL, 3 as GP, 1 as PL and 1 as SL), 33 biomarkers of MetR diet (1 identified as GL, 6 as GP, 4 as SP and 1 as SL) (Supplementary Figure S3A), and 7 biomarkers of healthy aging (2 identified as GL and 1 as PL).

Changes in the fatty acid profile of total lipids in the aged frontal cortex were even more limited than in the cerebellum. Thus, only a decreased content of 24:6n-3 (19%), decreased delta-6 desaturase activity (24%), and increased peroxisomal beta-oxidation (23%) in the aged frontal cortex were observed (Table 6 and Table 7). No additional changes were detected, including the oxidation-derived protein damage markers, in the aged frontal cortex compared to the young group (Table 6). MetR diet in old age neither modified nor introduced additional modifications to the minor changes verified in the aged group (Table 6, Table 7 and Table 8).

## 3. Discussion

Lipids are key components of brain structure that play a crucial role in main brain functions. However, there is a lack of studies analyzing the effects of aging and anti-aging interventions applied in old age on lipid composition in distinct regions of the rat brain, and using a lipidomics approach. The rat frontal cortex and cerebellum are brain regions that participate in motor and cognitive functions. Interestingly, these region-dependent functions can also be extended to humans, suggesting a functional unit across mammalian phylogeny. Consequently, it may be hypothesized that the molecular bases of the aging process and potential changes in the lipidome may be common between species, thus converting rodents into a good experimental model to extrapolate mechanisms of brain aging in humans [19,31,32,33,34]. In this context, in our study, a comprehensive lipidomic analysis of the rat frontal cortex and cerebellum was performed to learn the age-related changes of lipids in these regions, as well as to assess the effect of an anti-aging intervention, methionine restriction, applied in old age in order to evaluate whether the effect verified at a young age in brains by this dietary intervention [20,21,22,23,24,25] can also be reproduced at old age.

In the healthy adult rat brain, it seems that some fatty acid traits are shared in a cross-regional way. Thus, the fatty acid average chain length (ACL) is maintained at about 18 carbon atoms, in both regions, and the main SFAs are 16:0 and 18:0, whereas for PUFA 18:1n-9, 20:4n-6, and 22:6n-3 are predominant. Interestingly, these findings are analogous to what is observed in human brain [35], suggesting the maintenance of basic rules in the fatty acid profile of the nervous system in, at least, mammalian species. This idea is not, however, contradictory to the existence of interregional differences. Thus, the rat cerebellum is more enriched in UFAs—with a predominance of MUFAs, followed by PUFAn-3 and, finally, PUFAn-6—compared to the frontal cortex. This profile determines that the cerebellum presents a higher PI and, consequently, greater vulnerability to lipid peroxidation than the frontal cortex does. In addition, the rat cerebellum shows a greater steady-state level of protein oxidative damage with respect to the frontal cortex, indicating higher oxidative conditions in the cerebellum. However, and surprisingly, the lipoxidation-derived protein damage (expressed by the MDAL marker) was lower in the cerebellum than in the frontal cortex, suggesting the existence of efficient protective mechanisms that may be mediated, paradoxically, by the PUFA 22:6n-3, which, despite a high oxidative potential, is also an indirect antioxidant mediator inducing the expression of antioxidant systems and related pathways [36]. This might explain the better protection of the effects of aging and neurodegenerative diseases classically attributed to the cerebellum [26,27].

Minor but significant changes were detected for the fatty acid profiles with aging, with more substantial changes observed in the cerebellum (increased MUFA and decreased PUFA contents) in contrast to the frontal cortex, which showed a more sustained composition throughout the adult lifespan of rats. These observations are in line with previous studies in rats [15], as well as humans [37,38,39,40], suggesting that the maintenance of a fatty acid profile throughout the adult lifespan is a key prerequisite to ensuring optimal neuronal integrity and, surely, brain structure and function. In this line, the steady-state levels of different protein damage markers are also sustained during adult life. Reinforcing this idea, alterations in lipid profiles and protein damage are associated with the onset and development of diverse neurodegenerative diseases such as Alzheimer’s [3,12,41].

Significant changes were also observed with a lipidomics approach. However, these changes are minor again since they represent around 10% (60 out of 665 lipid species for cerebellum, and 81 out of 763 for frontal cortex) of the detected lipidome for both rat brain regions during aging, but with opposing changes in some cases between regions, and with a partial and reversible effect derived from MetR. Globally, the cerebellum seems to be more affected by the aging process, whereas the frontal cortex shows more changes according to diet applied. Remarkably, the most affected lipid classes were, for both regions, ether-triacylglycerols, diacylglycerols, phosphatidylethanolamine N-methylated, alkenyl-PE (plasmalogens), ceramides, and cholesterol esters. The observed changes in these lipid classes require special attention because the metabolic pathways and cell mechanisms behind them can be crucial in brain aging.

There are four functional categories associated with the different lipid classes identified: biosynthesis of membrane structural components, bioenergetics, antioxidant protection, and bioactive lipids. Thus, diacylglycerols and ceramides are components of cell membranes (specifically located in lipid rafts) and lipid mediators that participate in the regulation of a broad diversity of cell mechanisms and pathways, including protein kinase activities, cytoskeletal organization, cell survival, autophagy, and control of neuronal communication, among several others [42,43,44,45]. Plasmalogens are structural components of cell membranes that also play a role in the formation and stability of lipid raft microdomains, as well as in diverse cell functions including cholesterol transport, membrane fusion events, and vesicular function [46,47], but they also have antioxidant properties [46] that help to maintain membrane integrity. This antioxidant property is probably also shared by ether-triacylglycerols (TG-O), but at the lipid droplet level; the presence of this lipid class in rat brain was identified for the first time in this work. Triacylglycerides and cholesteryl esters are bioenergetic compounds that compose the lipid droplets, and they are also present in neural cells [48]. It is in this context we propose that the presence of ether-triacylglycerides in lipid droplets has an antioxidant property that preserves the integrity of this fat storage organelle. Finally, phosphatidylethanolamine N-methylated is a precursor for biosynthesis of phosphatidylcholine, a structural component in cell membranes, in a reaction mediated by phosphatidylethanolamine-N-methyltransferases, which uses metabolites generated in the methionine cycle as a substrate [49].

The observed changes during aging in different lipid species suggest the involvement, to a greater or lesser extent, of specific cell functions related to membrane structure, bioenergetics, antioxidant defense, and cell signaling. Both regions, the cerebellum and frontal cortex, share loss of antioxidant potential, deterioration in bioenergetic systems, increased ceramide content, and defects in the diacylglycerol-phosphatidic acid signaling pathway. In contrast, aging also differentially affects the cerebellum and frontal cortex, with the biosynthesis of phosphatidylcholine from the methylation of phosphatidylethanolamine (increased in cerebellum, but decreased in frontal cortex) and the cholesteryl ester content (decreased in cerebellum, and increased in frontal cortex) being the main interregional dissociation found. Methionine restriction, as an anti-aging intervention applied in old age, partially reverses changes induced by aging. Thus, in the cerebellum MetR seems to reverse changes in glycerolipids (especially ether-triacylglycerols), plasmalogens, and phosphatidylethanolamine N-methylated; whilst in the frontal cortex, MetR preferentially also affects phophatidylethanolamine N-methylated, ceramides, and cholesteryl esters.

Metabolite identification is still the bottleneck of LC-MS-based metabolomics/lipidomic studies and has some limitations [50]. Public databases are still incomplete, and several compounds do not have an associated MS/MS spectrum. Furthermore, most of the compound MS/MS spectra available are predicted. In our study, 90% of annotated compounds matched two or more criteria according to the Metabolomics Standards Initiative [29], but most of them (60%) are annotated based on exact mass and retention time because there is no available MS/MS spectrum in public databases.

We may conclude that the rat cerebellum and frontal cortex have efficient mechanisms to preserve most lipid profiles of their cell membranes throughout their adult lifespan in order to maintain brain structure and function, and that part of the small changes that take place during aging may be partially reversed with methionine restriction applied in old age.

## 4. Materials and Methods

### 4.1. Chemicals

Unless otherwise specified, all reagents were from Sigma-Aldrich (Madrid, Spain) and UPLC or LC/MS, purity grade.

### 4.2. Animals and Diets

Thirty male Wistar rats, obtained from Charles River, were individually caged and maintained in a 12:12 (light:dark) cycle at 22 ± 2 °C and 50 ± 10% relative humidity and separated into three groups: Adult, Aged, and Aged+MetR. Eight-month-old adult group (554.05 ± 17.36 g body weight) and 26-month-old Aged group (568.57 ± 38.92 g body weight) were fed a control diet, whereas the 26-month-old aged+MetR group (475.04 ± 21.30 g body weight) was fed an 80% L-methionine restricted diet. Semipurified diets were specifically prepared after our request by MP Biochemicals (Irvine, CA, USA) and were imported to Spain by Leti (Barcelona, Spain). Adult controls and Old controls received the control diet, and the Old-MetR group received the 80% MetR diet. The composition of the control diet (in g/100 g of diet) was L-arginine 1.12%, L-lysine 1.44%, L-histidine 0.33%, L-leucine 1.11%, L-isoleucine 0.82%, L-valine 0.82%, L-threonine 0.82%, L-tryptophan 0.18%, L-methionine 0.86%, L-glutamic acid 2.70%, L-phenylalanine 1.16%, L-glycine 2.33%, Dextrine 5.0%, corn starch 31.82%, sucrose 31.79%, cellulose 5.0%, choline bitartrate 0.20%, MP vitamin diet fortification mixture 1.0%, mineral mix (AIN) 3.50%, and corn oil 8.0%. The composition of the 80% MetR diet was similar to that of the control diet except that L-methionine was present at 0.17%, which corresponds to an amount of this amino acid 80% lower than in the control diet (0.86%). This 0.69% decrease in L-methionine in the 80% MetR diet was compensated by increasing all the rest of the dietary components in proportion to their presence in the diet [51]. The two control groups received the same amounts of food every day as the MetR group had eaten, on average, the previous week (pair feeding). After 7 weeks of MetR, the animals were sacrificed by decapitation. The frontal cortex and cerebellum samples were immediately frozen at −80 °C for subsequent analysis.

### 4.3. Sample Homogenization

The starting number of animals was 10 individuals per group. Since there was not enough tissue from all animals to do all the analyses, analyses were performed using a minimum of 7 samples per tissue. The type of analysis and grouping ensured sufficient statistical power. Tissue homogenization was performed on different subsamples from each brain region to guarantee they were as fresh as possible.

To do so, 50 mg of tissue was homogenized using a digital ULTRA-TURRAX^®^ instrument (IKA, Staufen, Germany) in a buffer containing 180 mM KCl, 5 mM MOPS, 2 mM EDTA, and 1 mM DTPAC adjusted to pH 7.4. Prior to homogenization, 1 μM BHT and a mix of protease (Sigma, Madrid, Spain) and phosphatase (1 mM Na3VO4, 1vM NaF) inhibitors were added. After a brief centrifugation of 1000 rpm for 3 min at 4 °C, the protein concentration was measured in the supernatants using the Bradford method (Bio-Rad Laboratories, Barcelona, Spain). A sample of 10 μL was used for the methyl tert-butyl ether (MTBE) extraction for liquid chromatography–mass spectrometry (LC-MS) lipidomic analysis, and the amount corresponding to 0.5 mg of protein was used for preparation of fatty acid methyl esters and analysis by gas chromatography with flame ionization detection (GC-FID).

### 4.4. Untargeted Lipidomic Analysis

Liquid chromatography–mass spectrometry based untargeted lipidomics was performed using an Agilent 1290 ultra-performance liquid chromatograph (UPLC) (Agilent Technologies, Barcelona, Spain) equipped with a Waters Acquity HSS T3 column (150 mm L, 2.1 mm ID, 1.8 µm particle size) (Waters, Milford, MA, USA) and coupled to an ESI-QTOF MS/MS 6520 model (Agilent Technologies, Barcelona, Spain), as previously described [24,52]. Total lipids from the rat frontal cortex and cerebellum samples were extracted with methyl tert-butyl ether (MTBE) [53] (see Supplementary Materials for more information). The list of internal lipid standards used and added to samples is indicated in Supplementary Materials. A fraction of all lipid extracts was pooled and used as quality controls.

Data were collected in positive and negative electrospray ionized (ESI) modes in a TOF mode, operated in full-scan mode at 100 to 3000 *m*/*z* in an extended dynamic range (2 GHz), using N_2_ as the nebulizer gas (5 L/min. 350 °C). The capillary voltage was set at 3500 V with a scan rate of 1 scan/s. Continuous infusion using a double spray with masses 121.050873, 922.009798 (positive ion mode) and 119.036320, 966.000725 (negative ion mode) was used for in-run calibration of the mass spectrometer.

Data were acquired using MassHunter Data Acquisition vB07.00 software (Agilent Technologies, Barcelona, Spain) and preprocessed using MassHunter Mass Profiler Professional software (Agilent Technologies, Barcelona, Spain), as previously described [54].

Before data processing, we checked each lipid standard’s relative abundances in all the samples for the quality assessment using specific software. All samples passed this quality assessment, supporting the robustness of the analyses. According to the specific metabolomics software, the intensity of features (measured as arbitrary units) was normalized using one representative internal standard (MassProfiler Professional) (Agilent Technologies, Barcelona, Spain). The sample with the highest internal standard abundance was used as a reference, and the other samples were scaled by multiplying the relative internal standard abundance of each sample compared to the reference. Class internal standards were used for retention time assessment. Only features found in at least 70% of the QC samples were taken into account to correct for individual bias. Instrumental drift was corrected using a LOESS approach [55,56]. The median (Q1–Q3) relative standard deviation for QC was 16% (7–43) in the cerebellum and 19% (7–34) in the frontal cortex. Multivariate statistics (principal component analysis (PCA), partial least squares-discriminant analysis (PLSDA), and hierarchical and classification analyses) were performed using Metaboanalyst v5.0 software (https://www.metaboanalyst.ca/).

Statistically differential molecules were determined using a Kruskal–Wallis test (*p* < 0.05) and annotated using the Human Metabolome Database v5.0 (https://hmdb.ca/) (exact mass ppm < 30 and adducts M+H, M+H-H2O, M+NH4, M+NH4-H2O, M+Na, M+K for positive ionization and M-H, M-H2O-H, M+CH3COO for negative ionization). According to a previously published paper [52], the established acceptable relative migration time for each lipid class was 3 min. MS/MS spectra were checked using the LipidMatch v3.5 (https://innovativeomics.com/software/lipidmatch-modular-annotates-a-feature-table/, accessed on 16 November 2021), Human Metabolome Database (https://hmdb.ca/, accessed on 16 November 2021) and MS DIAL (http://prime.psc.riken.jp/compms/msdial/main.html, accessed on 16 November 2021) software (see Supplementary Materials for more information).

### 4.5. Fatty Acid Profile

Fatty acids of total lipids from the rat cerebellum and frontal cortex homogenates were analyzed as methyl ester derivatives (FAMEs) by gas chromatography (GC), as previously described [41] (see Supplementary Materials for more information). Separation was performed with a DBWAX capillary column (30 m × 0.25 mm × 0.20 μm) in a GC System 7890A with a Series Injector 7683B and an FID detector (Agilent Technologies, Barcelona, Spain). Identification of fatty acid methyl esters was made by comparison with authentic standards (Larodan Fine Chemicals, Malmö, Sweden). Results are expressed as mol%.

The following fatty acyl indices were also calculated: saturated fatty acids (SFA); unsaturated fatty acids (UFA); monounsaturated fatty acids (MUFA); polyunsaturated fatty acids (PUFA) from n-3 and n-6 series (PUFAn-3 and PUFAn-6); and average chain length (ACL) = [(Σ% Total 14 × 14) + (Σ% Total 16 × 16) + (Σ% Total 18 × 18) + (Σ%Total 20 × 20) + (Σ% Total 22 × 22) + (Σ% Total 24 × 24)]/100. The density of double bonds was calculated with the double bond index, DBI = [(1 × Σ mol% monoenoic) + (2 × Σ mol% dienoic) + (3 × Σ mol% trienoic) + (4 × Σ mol% tetraenoic) + (5 × Σ mol% pentaenoic) + (6 × Σ mol% hexaenoic)]. The membrane susceptibility to peroxidation was calculated with the peroxidizability index, PI = [(0.025 × Σ mol% monoenoic) + (1 × Σ mol% dienoic) + (2 × Σ mol% trienoic) + (4 × Σ mol% tetraenoic) + (6 × Σ mol% pentaenoic) + (8 × Σ mol% hexaenoic)].

Elongase and desaturase activities were estimated from specific product/substrate ratios: D9D (n-7) = 16:1n-9/16:0; D9D (n-9) = 18:1n-9/18:0; D5D (n-6) = 20:4n-6/20:3n-6; D6D (n-3) (a) = 18:4n-3/18:3n-3; D6D (n-3) (b) = 24:6n-3/24:5n-3; Elovl3 (n-9) = 20:1n-9/18:1n-9; Elovl6 = 18:0/16:0; Elovl1-3-7 (a) = 20:0/18:0; Elovl1-3-7 (b) = 22:0/20:0; Elovl1-3-7 (c) = 24:0/22:0; Elovl5 (n-6) = 20:2n-6/18:2n-6; Elovl2-5 (n-6) = 22:4n-6/20:4n-6; Elovl2-5 (n-3) = 22:5n-3/20:5n-3, and Elovl2 (n-3) = 24:5n-3/22:5n-3. Finally, peroxisomal β-oxidation was estimated, although only few fatty acid species were measured, according to the ratio 22:6n-3/24:6n-3.

All values were expressed as means ± standard error of the mean (SEM). Comparisons between groups were analyzed with ANOVA followed by DMS tests for paired groups using the RStudio statistical software v3 (RStudio, Inc., Boston, MA, USA). The minimum level of statistical significance was set at *p* < 0.05 for all the analyses.

### 4.6. Markers of Mitochondrial Stress and Tissue Protein Damage

Different specific markers of protein oxidation (the protein carbonyl glutamic semialdehyde [GSA]), glycoxidation (carboxyethyl-lysine [CEL]), lipoxidation (malondialdehyde-lysine [MDAL]), and mixed glyco- and lipoxidation (carboxymethyl-lysine [CML]), as well as the marker of mitochondrial stress 2-S-(2-succino)cysteine [2-SC], were determined with gas chromatography–mass spectrometry (GC-MS) in samples from the rat cerebellum and frontal cortex, as previously described [41]. Protein damage markers were determined as trifluoroacetic acid methyl ester (TFAME) derivatives using an HP6890 Series II gas chromatograph (Agilent Technologies, Barcelona, Spain) with an Rtx-5MS Restek column (30 m × 0.25 mm × 0.25 μm) coupled to an MSD5973A Series detector, and the described temperature program [41]. Analyses were carried out by selected ion-monitoring GC/MS (SIM-GC/MS). The ions used were lysine and [2H8] lysine, *m*/*z* 180 and 187, respectively; 5-hydroxy-2-aminovaleric acid and [2H5] 5-hydroxy-2-aminovaleric acid (stable derivatives of GSA), *m*/*z* 280 and 285, respectively; CEL and [2H4] CEL, *m*/*z* 379 and 383, respectively; MDAL and [2H8] MDAL, *m*/*z* 474 and 482, respectively; CML and [2H2] CML, *m*/*z* 392 and 394, respectively; and 2-SC and [2H2] SC, *m*/*z* 284 and 286, respectively. The amounts of product were expressed as μmoles of the protein damage marker per mole of lysine.

All values were expressed as means ± standard error of the mean (SEM). Comparisons between groups were analyzed with ANOVA followed by DMS tests for paired groups using the RStudio statistical software v3 (RStudio, Inc., Boston, MA, USA). The minimum level of statistical significance was set at *p* < 0.05 for all the analyses.

## Figures and Tables

**Table 1:** Lipid species significantly different between experimental groups in the rat cerebellum.

Class	ID	Post Hoc	Biological Meaning (Biomarker)
GL	TG (52:0) ^a^	A < R	
	TG (47:0) ^b^	A < R	
	TG-O (54:1) ^b^	Ag, R < A	aging
	TG-O (60:1) ^b^	Ag, R < A	aging
	TG-O (47:0) ^b^	A, Ag < R	MetR
	TG-O (56:1) ^b^	Ag < R, A	healthy aging
GP	PS (36:1) ^a^	A < R	
	PE-P (38:4) ^a^	A < R	
	PG-P (38:5) ^b^	A < Ag, R	aging
	PE-P (40:4)/PE-O (40:5) ^a^	Ag, R < A	aging
	PA (44:7) ^a^	A < Ag, R	aging
	PE-Nme (36:6) ^b^	A < Ag, R	aging
	PC-O (38:4)/PC-P (38:3) ^b^	A < Ag, R	aging
	PE-Nme (34:3)/PE-NMe2 (33:3)/PC (32:3)/PE (35:3) ^b^	A, Ag < R	MetR
	PC (36:4)/PC-O (35:4) ^a^	A, Ag < R	MetR
	PS (40:4) ^a^	Ag < R, A	healthy aging
SP	Cer (34:1) ^a^	Ag < R, A	healthy aging
	Cer (38:4) ^a^	A < Ag, R	aging
	SM (33:1) ^b^	A < Ag, R	aging
	Ganglioside GA2 (44:1) ^b^	Ag, R < A	aging
	N-(2R-Hydroxyhexadecanoyl)-2S-amino-9-methyl-4E,8E-octadecadiene-1,3R-diol ^b^	A < Ag, R	aging
SL	CE (22:4) ^b^	Ag, R < A	aging
	cholest-5-en-3b-yl (11Z,14Z-eicosadienoate) ^c^	A, Ag < R	MetR
	CE (xx)	Ag < R, A	healthy aging

All compounds are putatively annotated compounds based upon physicochemical properties and/or spectral similarity with public/commercial spectral libraries [29]. ^a^ Identity (ID) based on exact mass, retention time (RT), and MS/MS spectrum; ^b^ ID based on exact mass and RT; ^c^ ID based on MS/MS spectrum. CE (xx): specific cholesteryl ester species could not be annotated, but its MS/MS spectrum displayed a CE characteristic pattern. GL: glycerolipids, GP: glycerophospholipids, SP: sphingolipids, SL: sterol lipids. Ag: Aged, R: Aged+MetR, A: Adult, n.i: no information. n (Adult) = 7, n (Aged) = 9, n (Aged+MetR) = 10.

**Table 2:** Fatty acid composition of total lipids in the rat cerebellum [30].

Fatty Acid	Adult	Aged	Aged+MetR
14:0	0.26 ± 0.00	0.29 ± 0.01 ^a,^*	0.32 ± 0.01 ^a,^***
16:0	17.93 ± 0.16	18.00 ± 0.04	17.83 ± 0.14
16:1n-7	0.38 ± 0.01	0.41 ± 0.02	0.40 ± 0.01
18:0	17.63 ± 0.06	17.70 ± 0.09	17.66 ± 0.08
18:1n-9	26.00 ± 0.14	26.53 ± 0.25	26.74 ± 0.21 ^a,^*
18:2n-6	1.14 ± 0.02	1.19 ± 0.04	1.37 ± 0.05 ^a,^***^; b,^**
18:3n-6	0.02 ± 0.00	0.02 ± 0.00	0.01 ± 0.00
18:3n-3	0.02 ± 0.00	0.02 ± 0.00	0.01 ± 0.00 ^a,^*
18:4n-3	0.03 ± 0.00	0.03 ± 0.00	0.03 ± 0.00
20:0	0.70 ± 0.04	0.83 ± 0.01 ^a,^*	0.79 ± 0.04
20:1n-9	4.06 ± 0.14	4.48 ± 0.07 ^a,^*	4.49 ± 0.10 ^a,^*
20:2n-6	0.33 ± 0.01	0.29 ± 0.01 ^a,^**	0.30 ± 0.01
20:3n-6	0.31 ± 0.01	0.30 ± 0.01	0.32 ± 0.01
20:4n-6	7.69 ± 0.08	7.27 ± 0.09 ^a,^**	7.36 ± 0.09 ^a,^*
20:5n-3	0.04 ± 0.00	0.04 ± 0.00 ^a,^*	0.04 ± 0.00
22:0	0.74 ± 0.02	0.75 ± 0.01	0.76 ± 0.02
22:1n-9	1.08 ± 0.09	1.01 ± 0.02	1.03 ± 0.03
22:4n-6	2.60 ± 0.03	2.22 ± 0.05 ^a,^***	2.22 ± 0.05 ^a,^***
22:5n-6	0.43 ± 0.03	0.45 ± 0.03	0.41 ± 0.03
22:5n-3	0.14 ± 0.00	0.14 ± 0.01	0.12 ± 0.00
22:6n-3	13.43 ± 0.12	12.96 ± 0.14	12.89 ± 0.17 ^a,^*
24:0	1.11 ± 0.05	1.00 ± 0.02	1.03 ± 0.02
24:1	2.26 ± 0.05	2.54 ± 0.10	2.41 ± 0.08
24:5n-3	0.04 ± 0.00	0.04 ± 0.00	0.04 ± 0.00
24:6n-3	1.31 ± 0.04	1.38 ± 0.03	1.45 ± 0.03 ^a,^*

Data are expressed as mean ± SEM. ^a^ Comparison between aged and aged+MetR with the adult group; ^b^ comparison between aged and aged+MetR groups. * *p* < 0.05. ** *p* < 0.01. *** *p* < 0.001. n (Adult) = 8, n (Aged) = 8, n (Aged+MetR) = 8.

**Table 3:** Calculated fatty acid indices from the fatty acid profile in the rat cerebellum.

Fatty Acid Index	Adult	Aged	Aged+MetR
ACL	18.91 ± 0.01	18.75 ± 0.08	18.89 ± 0.00
SFA	38.37 ± 0.17	38.35 ± 0.16	38.24 ± 0.14
UFA	61.52 ± 0.15	61.41 ± 0.12	61.58 ± 0.20
MUFA	33.64 ± 0.35	35.27 ± 0.16 ^a,^**	35.01 ± 0.33 ^a,^**
PUFA	27.53 ± 0.19	26.05 ± 0.19 ^a,^***	26.57 ± 0.16 ^a,^**
PUFAn-3	15.00 ± 0.10	14.59 ± 0.16	14.58 ± 0.15
PUFAn-6	12.53 ± 0.12	11.65 ± 0.12 ^a,^***	11.99 ± 0.15 ^a,^*
DBI	170.56 ± 0.96	165.22 ± 0.86 ^a,^***	166.91 ± 0.64 ^a,^*
PI	166.05 ± 1.14	158.66 ± 1.08 ^a,^***	160.06 ± 1.27 ^a,^**
D9D (n-7)	0.02 ± 0.00	0.02 ± 0.00	0.02 ± 0.00
D9D (n-9)	1.47 ± 0.01	1.50 ± 0.02	1.51 ± 0.02
D5D (n-6)	24.65 ± 0.42	24.45 ± 0.73	23.36 ± 0.63
D6D (n-3) (a)	1.84 ± 0.05	1.83 ± 0.08	1.99 ± 0.09
D6D (n-3) (b)	31.22 ± 1.76	34.49 ± 1.58	36.78 ± 1.87
Elovl3 (n-9)	0.16 ± 0.01	0.17 ± 0.00	0.17 ± 0.00
Elovl6	0.98 ± 0.01	0.98 ± 0.00	0.99 ± 0.01
Elovl1-3-7 (a)	0.04 ± 0.00	0.05 ± 0.00 ^a,^*	0.04 ± 0.00
Elovl1-3-7 (b)	1.09 ± 0.05	0.91 ± 0.01 ^a,^**	0.97 ± 0.04
Elovl1-3-7 (c)	1.49 ± 0.06	1.34 ± 0.03 ^a,^*	1.33 ± 0.01 ^a,^*
Elovl5 (n-6)	0.29 ± 0.01	0.23 ± 0.00 ^a,^***	0.22 ± 0.00 ^a,^***
Elovl2-5 (n-6)	0.34 ± 0.00	0.31 ± 0.01 ^a,^***	0.30 ± 0.01 ^a,^***
Elovl2-5 (n-3)	3.76 ± 0.07	3.30 ± 0.18 ^a,^*	3.11 ± 0.12 ^a,^**
Elovl2 (n-3)	0.31 ± 0.01	0.30 ± 0.01	0.34 ± 0.01
Peroxisomal β-oxidation	10.39 ± 0.44	9.42 ± 0.15	8.91 ± 0.28 ^a,^**

Abbreviations: ACL, average chain length; SFA, saturated fatty acids; UFA, unsaturated fatty acids; MUFA, monounsaturated fatty acids; PUFA, polyunsaturated fatty acids, and from n-3 and n-6 series (PUFAn-3 and PUFAn-6). Calculations: ACL = [(Σ% Total 14 × 14) + (Σ% Total 16 × 16) + (Σ% Total 18 × 18) + (Σ% Total 20 × 20) + (Σ% Total 22 × 22) + (Σ% Total 24 × 24)]/100. The density of double bonds in the membrane was calculated with the Double Bond Index. DBI = [(1 × Σ mol% monoenoic) + (2 × Σ mol% dienoic) + (3 × Σ mol% trienoic) + (4 × Σ mol% tetraenoic) + (5 × Σ mol% pentaenoic) + (6 × Σ mol% hexaenoic)]. Membrane susceptibility to peroxidation was calculated with the Peroxidizability Index. PI= [(0.025 × Σ mol% monoenoic) + (1 × Σ mol% dienoic) + (2 × Σ mol% trienoic) + (4 × Σ mol% tetraenoic) + (6 × Σ mol% pentaenoic) + (8 × Σ mol% hexaenoic)]. Elongase and desaturase activities were estimated from specific product/substrate ratios: delta-9-Desaturase (D9D) (n-7) = 16:1n-9/16:0; delta-9-Desaturase (D9D) (n-9) = 18:1n-9/18:0; delta-5-Desaturase (D5D) (n-6) = 20:4n-6/20:3n-6; delta-6-Desaturase (D6D) (n-3) (a) = 18:4n-3/18:3n-3; delta-6-Desaturase (D6D) (n-3) (b) = 24:6n-3/24:5n-3; Elovl3 (n-9) = 20:1n-9/18:1n-9; Elovl6 = 18:0/16:0; Elovl1-3-7 (a) = 20:0/18:0; Elovl1-3-7 (b) = 22:0/20:0; Elovl1-3-7 (c) = 24:0/22:0; Elovl5 (n-6) = 20:2n-6/18:2n-6; Elovl2-5 (n-6) = 22:4n-6/20:4n-6; Elovl2-5 (n-3) = 22:5n-3/20:5n-3, and Elovl2 (n-3)= 24:5n-3/22:5n-3. Finally, peroxisomal β-oxidation was estimated according to the ratio 22:6n-3/24:6n-3. Data are expressed as mean ± SEM. ^a^ comparison between aged and aged+MetR with the adult group. * *p* < 0.05, ** *p* < 0.01, *** *p* < 0.001. n (Adult) = 8, n (Aged) = 8, n (Aged+MetR) = 8.

**Table 4:** Oxidation-derived protein damage markers in the rat cerebellum.

Damage Marker	Adult	Aged	Aged+MetR
GSA	5013.14 ± 144.38	4376.78 ± 116.83 ^a,^*	4410.87 ± 77.14 ^a,^*
CEL	579.82 ± 39.05	725.42 ± 93.56	660.52 ± 37.93
CML	1408.30 ± 59.83	1387.38 ± 105.08	1302.08 ± 43.07
MDAL	196.20 ± 8.66	179.29 ± 5.01	195.32 ± 8.66
2-SC	39.79 ± 4.34	26.85 ± 1.80 ^a,^*	26.79 ± 2.27 ^a,^*

Abbreviations: GSA, glutamic semialdehyde; CEL, carboxyethyl-lysine; CML, carboxymethyl-lysine; MDAL, malondialdehyde lysine; 2-SC, 2-succinyl-cysteine. Data are expressed as mean ± SEM. ^a^ comparison between aged and aged + MetR with the adult group. * *p* < 0.05. Units: µmol/mol lysine. n (Adult) = 8, n (Aged) = 8, n (Aged+MetR) = 8.

**Table 5:** Lipid species significantly different between experimental groups in the rat frontal cortex.

Class	ID	Post Hoc	Biological Meaning (Biomarker)
FA	12-hydroxyheptadecanoic acid ^b^	Ag, R < A	aging
GL	TG (50:0) ^b^	R < A	
	TG (48:1) ^b^	Ag, R < A	aging
	TG (54:6) ^b^	Ag, R < A	aging
	TG (50:1) ^b^	Ag, R < A	aging
	TG (64:14) ^b^	A < Ag, R	aging
	TG (47:0) ^b^	Ag < R	
	TG-O (58:10) ^a^	R < Ag	
	DG (40:3) ^b^	R < Ag	
	TG (64:14) ^a^	A, Ag < R	MetR
	TG-O (60:9) ^b^	R, A < Ag	healthy aging
	TG (62:12) ^b^	Ag < R, A	healthy aging
GP	PE (36:1) ^a^	R < A	
	PE (39:5)/PE-P (40:4) ^a^	R < A	
	PC (36:2) ^a^	Ag, R < A	aging
	PE-P (40:6) ^a^	Ag, R < A	aging
	PE-P (38:6) ^a^	Ag, R < A	aging
	PE-NMe (34:3)/PE (35:4)/PC (32:3) ^b^	A, Ag < R	MetR
	PE-NMe (34:3)/PC (32:3) ^b^	A, Ag < R	MetR
	PA (44:7) ^a^	A, Ag < R	MetR
	PE (40:4) ^a^	R < A, Ag	MetR
	PE (38:1) ^a^	R < A, Ag	MetR
	PE-P (38:1) ^a^	R < A, Ag	MetR
	PI (38:4) ^a^	Ag < R, A	healthy aging
	PI (38:5) ^a^	Ag < A	
	PG (34:1) ^a^	Ag < R, A	healthy aging
PL	Dolichol-20 ^d^	A < Ag, R	aging
SP	Cer (36:1) ^a^	Ag < A	
	LacCer (34:2)/GalCer (44:2) ^b^	R < A, Ag	MetR
	Cer (36:1) ^b^	R < A, Ag	MetR
	N-(2R-Hydroxyhexadecanoyl)-2S-amino-9-methyl-4E,8E-octadecadiene-1,3R-diol ^b^	R < A, Ag	MetR
	Cer (34:1) ^c^	R < A, Ag	MetR
SL	CE (20:4) ^b^	A < Ag, R	aging
	CE (5:0) ^b^	A, Ag < R	MetR

All compounds are putatively annotated compounds based upon physicochemical properties and/or spectral similarity with public/commercial spectral libraries [29]. ^a^ Identity (ID) based on exact mass, retention time (RT), and MS/MS spectrum; ^b^ ID based on exact mass and RT; ^c^ ID based on MS/MS spectrum; (d) ID based on exact mass. FA: fatty acyls, GL: glycerolipids, GP: glycerophospholipids, SP: sphingolipids, SL: sterol lipids. Ag: Aged, R: Aged+MetR, A: Adult, n.i: no information. n (Adult) = 7, n (Aged) = 7, n (Aged+MetR) = 7.

**Table 6:** Fatty acid composition of total lipids in the rat frontal cortex [30].

Fatty Acid	Adult	Aged	Aged+MetR
14:0	0.92 ± 0.02	0.91 ± 0.01	0.92 ± 0.02
16:0	29.03 ± 0.014	29.31 ± 0.26	28.83 ± 0.12
16:1n-7	0.54 ± 0.02	0.56 ± 0.03	0.52 ± 0.01
18:0	24.17 ± 0.23	24.19 ± 0.11	24.20 ± 0.10
18:1n-9	19.71 ± 0.26	19.18 ± 0.19	19.47 ± 0.20
18:2n-6	0.94 ± 0.02	1.00 ± 0.03	1.06 ± 0.06
18:3n-6	0.03 ± 0.00	0.04 ± 0.00	0.04 ± 0.00
18:3n-3	0.03 ± 0.00	0.04 ± 0.00	0.04 ± 0.00
18:4n-3	0.04 ± 0.00	0.04 ± 0.00	0.05 ± 0.00
20:0	0.18 ± 0.01	0.18 ± 0.01	0.19 ± 0.01
20:1n-9	0.71 ± 0.04	0.68 ± 0.04	0.71 ± 0.04
20:2n-6	0.11 ± 0.00	0.11 ± 0.00	0.12 ± 0.01
20:3n-6	0.20 ± 0.00	0.22 ± 0.01	0.24 ± 0.01 ^a,^***
20:4n-6	10.20 ± 0.26	10.49 ± 0.11	10.53 ± 0.18
20:5n-3	0.04 ± 0.00	0.04 ± 0.00	0.04 ± 0.00
22:0	0.18 ± 0.01	0.16 ± 0.01	0.16 ± 0.00
22:1n-9	0.57 ± 0.05	0.58 ± 0.03	0.62 ± 0.02
22:4n-6	2.38 ± 0.08	2.35 ± 0.05	2.35 ± 0.07
22:5n-6	0.80 ± 0.07	0.99 ± 0.09	0.91 ± 0.08
22:5n-3	0.09 ± 0.00	0.10 ± 0.00	0.10 ± 0.00
22:6n-3	7.95 ± 0.25	8.31 ± 0.19	8.36 ± 0.17
24:0	0.15 ± 0.01	0.17 ± 0.00	0.17 ± 0.01
24:1	0.17 ± 0.01	0.14 ± 0.01	0.14 ± 0.01
24:5n-3	0.04 ± 0.00	0.04 ± 0.00	0.04 ± 0.00
24:6n-3	0.19 ± 0.01	0.15 ± 0.00 ^a,^*	0.15 ± 0.01 ^a,^**

Data are expressed as mean ± SEM. ^a^ Comparison between aged and aged+MetR with the adult group. * *p* < 0.05, ** *p* < 0.01, *** *p* < 0.001. n (Adult) = 8, n (Aged) = 8, n (Aged+MetR) = 8.

**Table 7:** Calculated indices from the fatty acid profile in the rat frontal cortex.

Fatty Acid Index	Adult	Aged	Aged+MetR
ACL	18.12 ± 0.01	18.13 ± 0.01	18.12 ± 0.02
SFA	54.63 ± 0.19	54.89 ± 0.35	54.45 ± 0.15
UFA	44.67 ± 0.58	45.09 ± 0.34	45.43 ± 0.16
MUFA	21.77 ± 0.33	21.15 ± 0.25	21.40 ± 0.27
PUFA	23.05 ± 0.58	23.94 ± 0.30	24.03 ± 0.33
PUFAn-3	8.57 ± 0.17	8.74 ± 0.18	8.78 ± 0.17
PUFAn-6	14.67 ± 0.36	15.20 ± 0.19	15.25 ± 0.27
DBI	128.67 ± 2.74	132.49 ± 1.52	132.94 ± 1.43
PI	123.50 ± 3.31	128.55 ± 1.91	128.69 ± 1.97
D9D (n-7)	0.02 ± 0.00	0.02 ± 0.00	0.02 ± 0.00
D9D (n-9)	0.82 ± 0.01	0.79 ± 0.01	0.80 ± 0.01
D5D (n-6)	49.68 ± 0.31	47.75 ± 2.05	43.81 ± 1.12 ^a,^*
D6D (n-3) (a)	1.03 ± 0.16	1.10 ± 0.07	1.18 ± 0.06
D6D (n-3) (b)	5.10 ± 0.55	3.88 ± 0.34 ^a,^*	3.86 ± 0.36
Elovl3 (n-9)	0.04 ± 0.00	0.04 ± 0.00	0.04 ± 0.00
Elovl6	0.83 ± 0.01	0.83 ± 0.00	0.84 ± 0.01
Elovl1-3-7 (a)	0.01 ± 0.00	0.01 ± 0.00	0.01 ± 0.00
Elovl1-3-7 (b)	1.02 ± 0.04	0.86 ± 0.03 ^a,^**	0.86 ± 0.02 ^a,^**
Elovl1-3-7 (c)	0.85 ± 0.05	1.01 ± 0.15	1.07 ± 0.08
Elovl5 (n-6)	0.12 ± 0.00	0.11 ± 0.00	0.11 ± 0.00
Elovl2-5 (n-6)	0.23 ± 0.00	0.22 ± 0.00	0.22 ± 0.01
Elovl2-5 (n-3)	2.76 ± 0.22	2.65 ± 0.15	2.43 ± 0.14
Elovl2 (n-3)	0.41 ± 0.03	0.40 ± 0.02	0.42 ± 0.03
Peroxisomal β-oxidation	43.85 ± 3.05	54.04 ± 2.12 ^a,^*	56.40 ± 1.92 ^a,^**

For details, see Table 3. Data are expressed as mean ± SEM. ^a^ comparison between aged and aged+MetR with the adult group. * *p* < 0.05, ** *p* < 0.01. n (Adult) = 8, n (Aged) = 8, n (Aged+MetR) = 8.

**Table 8:** Oxidation-derived protein damage markers in the rat frontal cortex.

Damage Marker	Adults	Aged	Aged+MetR
GSA	3615.40 ± 79.03	3700.19 ± 138.23	3623.83 ± 246.00
CEL	270.70 ± 15.58	259.79 ± 29.33	233.04 ± 11.36
CML	966.24 ± 40.79	1016.19 ± 63.22	949.69 ± 85.01
MDAL	372.59 ± 30.48	345.74 ± 6.80	376.28 ± 21.86
2-SC	28.43 ± 4.18	20.78 ± 2.28	35.55 ± 4.99 ^b,^*

For details, see Table 4. Data are expressed as mean ± SEM. ^b^ comparison between aged and aged+MetR groups. * *p* < 0.05. Units: µmol/mol lysine. n (Adult) = 7, n (Aged)= 8, n (Aged+MetR) = 8.

## Data Availability

The datasets generated and/or analyzed during the current study are available from the corresponding author on reasonable request.

## Supplementary Materials

The following are available online at https://www.mdpi.com/article/10.3390/ijms222212517/s1.
