# Supplementary material for: Age-Related Changes in Lipidome of Rat Frontal Cortex and Cerebellum Are Partially Reversed by Methionine Restriction Applied in Old Age
Source: Int J Mol Sci. 2021 Nov 20;22(22):12517. doi: 10.3390/ijms222212517 (PMC8623997; doi:10.3390/ijms222212517)
Supplement: Supplementary file 1 [file ijms-22-12517-s001.zip › ijms-1450261-Supplementary DataSet.pdf]

Cerebellum

Class	ID	m/z	RT	Relative migration time	RT class authentic standard	ionization	p-value	post hoc	Biological Meaning (biomarker)
GL	TG(52:0)[a]	880,8451	10,6	-0,2	10,4	positive	0,024	A < R	
	TG(47:0)[b]	810,7604	10,1	0,3	10,4	positive	0,038	A < R	
	TG-O(54:1)[b]	857,8546	10,4	0	10,4	positive	0,002	Ag, R < A	aging
	TG-O(60:1)[b]	941,9425	10,4	0	10,4	positive	0,02	Ag, R < A	aging
	TG-O(47:0)[b]	796,7588	10,1	0,3	10,4	positive	0,037	A, Ag < R	MetR
	TG-O(56:1)[b]	885,877	10,4	0	10,4	positive	0,046	Ag < R, A	healthy aging
GP	PS(36:1)[a]	770,5363	7,9	0,4	8,3	negative	0,012	A < R	
	PE-P(38:4)[a]	750,512	7,8	-0,6	7,2	negative	0,05	A < R	
	PG-P(38:5)[b]	857,4811	6	1,9	7,9	negative	0,009	A < Ag, R	aging
	PE-P(40:4)/PE-O(40:5)[a]	780,6043	8	-0,8	7,2	positive	0,022	Ag, R < A	aging
	PA(44:7)[a]	801,5366	9,2	-1,5	7,7	negative	0,032	A < Ag, R	aging
	PE-Nme(36:6)[b]	748,4965	7,3	-0,1	7,2	negative	0,036	A < Ag, R	aging
	PC-O(38:4)/PC-P(38:3)b	854,6343	8,3	-0,6	7,7	negative	0,043	A < Ag, R	aging
	PE-Nme(34:3)/PE-NMe2(33:3)/PC(32:3)/PE(35:3)[b]	708,5005	8,8	-1,6	7,2	negative	0,021	A, Ag < R	MetR
	PC(36:4)/PC-O(35:4)a	754,5605	7	0,7	7,7	positive	0,041	A, Ag < R	MetR
	PS(40:4)[a]	840,5795	6,8	1,5	8,3	positive	0,041	Ag < R, A	healthy aging
SP	Cer(34:1)[a]	538,5565	7,1	0,5	7,6	positive	0,024	Ag < R, A	healthy aging
	Cer(38:4)[a]	646,5284	9,4	-1,8	7,6	negative	0,01	A < Ag, R	aging
	SM(33:1)[b]	687,5467	9,2	-2,5	6,7	negative	0,02	A < Ag, R	aging
	Ganglioside GA2 (44:1)[b]	1222,874	7,2	n.i.	n.i.	positive	0,04	Ag, R < A	aging
	N-(2R-Hydroxyhexadecanoyl)-2S-amino-9-methyl-4E,8E-octadecadiene-1,3R-diol[b]	564,5108	7,9	n.i.	n.i.	negative	0,043	A < Ag, R	aging
SL	CE(22:4)[b]	700,6393	9,8	0,9	10,7	positive	0,042	Ag, R < A	aging
	cholest-5-en-3b-yl (11Z,14Z-eicosadienoate)[c]	675,6354	8,4	n.i.	n.i.	negative	0,045	A, Ag < R	MetR
	CE(xx)	705,594	9,9	0,8	10,7	positive	0,037	Ag < R, A	healthy aging
Unknown	936.5373@6.268	936,5373	6,3			negative	0,005	A < R	
	2164.604@10.817	2164,604	10,8			positive	0,032	R < A	
	1255.994@8.785	1255,994	8,8			negative	0,033	A < R	
	1087.208@3.454	1087,208	3,5			positive	0,035	R < A	
	777.7811@10.122	777,7811	10,1			positive	0,048	A < R	
	878.8967@10.119	878,8967	10,1			positive	0,031	A < Ag	
	1168.847@0.924	1168,847	0,9			positive	0,043	A < Ag	
	112.993@0.850	112,993	0,9			negative	<0.001	A < Ag, R	aging
	307.331@0.919	307,3311	0,9			positive	<0.001	Ag, R < A	aging
	241.123@0.804	241,123	0,8			negative	0,001	Ag, R < A	aging
	285.268@4.349	285,2688	4,3			negative	0,002	A < Ag, R	aging
	213.157@0.885	213,1578	0,9			negative	0,005	Ag, R < A	aging
	1086.804@8.148	1086,804	8,1			negative	0,019	A < Ag, R	aging
	2238.622@10.851	2238,622	10,9			positive	0,019	Ag, R < A	aging
	861.609@8.144	861,6093	8,1			negative	0,02	A < Ag, R	aging
	513.151@3.456	513,1511	3,5			positive	0,021	Ag, R < A	aging
	1353.093@10.945	1353,093	10,9			positive	0,026	A < Ag, R	aging
	853.260@6.622	853,2602	6,6			positive	0,027	A < Ag, R	aging
	1697.653@10.405	1697,653	10,4			positive	0,034	Ag, R < A	aging

656.223@4.218	656,2233	4,2	positive	0,043	A < Ag, R	aging
835.250@8.984	835,2506	9	positive	0,023	Ag < R	
2016.566@10.742	2016,566	10,7	positive	0,037	R < Ag	
1674.505@10.468	1674,505	10,5	positive	0,038	R < Ag	
1646.467@10.472	1646,467	10,5	positive	0,006	R < A, Ag	MetR
1119.182@3.461	1119,182	3,5	positive	0,008	R < A, Ag	MetR
2090.582@10.782	2090,582	10,8	positive	0,019	R < A, Ag	MetR
536.5306@9.686	536,5306	9,7	negative	0,02	A, Ag < R	MetR
376.3955@10.698	376,3955	10,7	positive	0,039	A, Ag < R	MetR
691.5533@9.0	691,5533	9	negative	0,037	A < Ag, R	aging
691.5118@8.8	691,5118	8,8	negative	0,029	A, Ag < R	MetR
1602.178@7.3	1602,178	7,3	positive	0,019	Ag, R < A	aging
1342.953@7.9	1342,953	7,9	positive	0,032	Ag < R	
697.607@2.890	697,6074	2,9	positive	<0.001	R < Ag < A	
966.6473@8.163	966,6473	8,2	negative	0,006	A, Ag < R	MetR
896.4409@7.3	896,4409	7,3	negative	<0.001	A < Ag, R	aging
930.6769@9.624	930,6769	9,6	negative	0,019	A < Ag, R	aging

Frontal cortex

Class	ID	m/z	RT	Relative migration time	RT class authentic standard	ionization	p-value	post hoc	Biological meaning (biomarker)
FA	12-hydroxyheptadecanoic acid[b]	304,289	2	2,1	4,1	positive	0,034	Ag, R < A	aging
GL	TG(50:0)[b]	852,809	10	0,4	10,4	positive	0,047	R < A	
	TG(48:1)[b]	822,769	10	0,4	10,4	positive	0,002	Ag, R < A	aging
	TG(54:6)[b]	896,782	9,8	0,6	10,4	positive	0,006	Ag, R < A	aging
	TG(50:1)[b]	850,798	10	0,4	10,4	positive	0,025	Ag, R < A	aging
	TG(64:14)[b]	1061,8	11	-0,6	10,4	negative	0,03	A < Ag, R	aging
	TG(47:0)[b]	810,758	10	0,4	10,4	positive	0,024	Ag < R	
	TG-O(58:10)[a]	911,762	8,3	2,1	10,4	negative	0,04	R < Ag	
	DG(40:3)[b]	706,631	9,2	-1	8,2	positive	0,043	R < Ag	
	TG(64:14)[a]	1001,76	11	0,6	10,4	negative	0,036	A, Ag < R	MetR
	TG-O(60:9)[b]	960,84	11	-0,6	10,4	positive	0,003	R, A < Ag	healthy aging
	TG(62:12)[b]	959,751	10	0,4	10,4	negative	0,032	Ag < R, A	healthy aging
GP	PE(36:1)[a]	744,52	7,9	-0,7	7,2	negative	0,04	R < A	
	PE(39:5)/PE-P(40:4)[a]	778,54	8,1	-0,9	7,2	negative	0,05	R < A	
	PC(36:2)[a]	844,568	7,6	0,1	7,7	negative	0,003	Ag, R < A	aging
	PE-P(40:6)[a]	776,575	7,6	-0,4	7,2	positive	0,018	Ag, R < A	aging
	PE-P(38:6)[a]	748,54	7,1	0,1	7,2	positive	0,041	Ag, R < A	aging
	PE-NMe(34:3)/ PE(35:4)/PC(32:3)[b]	708,497	8,8	-1,6	7,2	negative	0,002	A, Ag < R	MetR
	PE-NMe(34:3)/PC(32:3)[b]	726,506	6,4	0,8	7,2	negative	0,007	A, Ag < R	MetR
	PA(44:7)[a]	801,533	9,2	-1,5	7,7	negative	0,027	A, Ag < R	MetR
	PE(40:4)[a]	794,534	7,8	-0,6	7,2	negative	0,028	R < A, Ag	MetR
	PE(38:1)[a]	772,551	8,3	-1,1	7,2	negative	0,045	R < A, Ag	MetR
	PE-P(38:1)[a]	756,555	8,5	-1,3	7,2	negative	0,05	R < A, Ag	MetR
	PI(38:4)[a]	885,509	6,5	1,6	8,1	negative	0,0295	Ag < R, A	healthy aging
	PI(38:5)[a]	883,492	6	2,1	8,1	negative	0,0297	Ag < A	
	PG(34:1)[a]	747,482	6,5	1,4	7,9	negative	0,044	Ag < R, A	healthy aging
PL	Dolichol-20[d]	1399,33	11	n.i.	n.i.	positive	0,049	A < Ag, R	aging
SP	Cer(36:1)[a]	566,555	7,7	-0,1	7,6	positive	0,037	Ag < A	
	LacCer(34:2)/GalCer(44:2)[b]	1058,77	7,8	-0,2	7,6	negative	0,004	R < A, Ag	MetR
	Cer(36:1)[b]	566,556	7,9	-0,3	7,6	positive	0,017	R < A, Ag	MetR
	N-(2R-Hydroxyhexadecanoyl)-2S-amino-9-methyl-4E,8E-octadecadiene-1,3R-diol[b]	548,507	8,5	n.i.	n.i.	positive	0,024	R < A, Ag	MetR
	Cer(34:1)[c]	536,527	9,7	-2,1	7,6	negative	0,033	R < A, Ag	MetR
SL	CE(20:4)[b]	690,626	10	0,7	10,7	positive	0,034	A < Ag, R	aging
	CE(5:0)[b]	529,438	8,5	2,2	10,7	negative	0,037	A, Ag < R	MetR
	1093.183@7.431	1093,18	7,4			negative	0,033	A < R	
	607.0378@4.049	607,038	4			positive	0,037	R < A	
	436.292@5.6	436,292	5,6			negative	0,007	A < R	
	697.609@2.9	697,609				positive	<0.001	R < A, Ag	MetR
	1613.475@10.402	1613,48	10			positive	0,038	R < A	

	1082.769@7.782	1082,77	7,8	negative	0,047	R < A	
	376.3928@10.701	376,393	11	positive	0,012	A < Ag	
	480.553@9.8	480,553	9,8	positive	0,006	Ag, R < A	aging
	1276.365@10.037	1276,37	10	positive	0,01	A < Ag, R	aging
	632.5244@8.285	632,524	8,3	negative	0,012	Ag, R < A	aging
	714.6518@8.229	714,652	8,2	positive	0,015	A < Ag, R	aging
	1618.478@10.658	1618,48	11	positive	0,016	Ag, R < A	aging
	680.653@7.405	680,653	7,4	negative	0,018	Ag, R < A	aging
	1074.864@7.612	1074,86	7,6	positive	0,02	Ag, R < A	aging
	1496.16@11.076	1496,16	11	positive	0,028	A < Ag, R	aging
	1363.26@10.575	1363,26	11	positive	0,031	A < Ag, R	aging
	1042.194@7.418	1042,19	7,4	negative	0,034	A < Ag, R	aging
	1995.934@10.699	1995,93	11	positive	0,035	Ag, R < A	aging
	751.8175@11.196	751,818	11	positive	0,038	Ag, R < A	aging
	1554.609@9.909	1554,61	9,9	positive	0,046	A < Ag, R	aging
	351.2056@3.366	351,206	3,4	negative	0,025	Ag < R	
	758.2307@8.673	758,231	8,7	positive	0,001	R < A, Ag	MetR
	610.1905@7.925	610,191	7,9	positive	0,001	R < A, Ag	MetR
**Unknown**	299.1883@2.437	299,188	2,4	negative	0,001	A, Ag < R	MetR
	926.4711@6.512	926,471	6,5	negative	0,001	A. Ag < R	MetR
	847.5224@9.222	847,522	9,2	negative	0,002	A, Ag < R	MetR
	590.1774@4.049	590,177	4	positive	0,002	R < A, Ag	MetR
	1168.847@0.925	1168,85	0,9	positive	0,003	R < A, Ag	MetR
	1151.151@4.052	1151,15	4,1	positive	0,004	R < A, Ag	MetR
	558.1972@3.454	558,197	3,5	positive	0,005	R < A, Ag	MetR
	684.2108@8.303	684,211	8,3	positive	0,006	R < A, Ag	MetR
	744.2671@7.070	744,267	7,1	positive	0,006	R < A, Ag	MetR
	1087.203@3.455	1087,2	3,5	positive	0,01	R < A, Ag	MetR
	691.9869@8.674	691,987	8,7	positive	0,013	A, Ag < R	MetR
	850.2615@6.596	850,262	6,6	positive	0,015	R < A, Ag	MetR
	635.5068@8.3	635,507	8,3	negative	0,029	Ag, R < A	aging
	761.2285@8.673	761,229	8,7	positive	0,015	R < A, Ag	MetR
	853.258@6.558	853,258	6,6	positive	0,02	R < A, Ag	MetR
	575.0669@3.448	575,067	3,4	positive	0,02	R < A, Ag	MetR
	878.8988@10.785	878,899	11	positive	0,022	A, Ag < R	MetR
	1169.351@9.792	1169,35	9,8	positive	0,031	R < A, Ag	MetR
	645.4989@8.799	645,499	8,8	negative	0,036	A, Ag < R	MetR
	656.2247@4.230	656,225	4,2	positive	0,004	R, A < Ag	healthy aging
	1019.752@10.248	1019,75	10	negative	0,012	Ag < R, A	healthy aging
	1353.087@10.945	1353,09	11	positive	0,022	R, A < Ag	healthy aging
	1586.176@7.6	1586,18	7,6	positive	0,039	Ag, R < A	aging
	1502.135@7.4	1502,14	7,4	positive	0,024	R < A	
