# Supplementary material for: Age-Related Changes in Lipidome of Rat Frontal Cortex and Cerebellum Are Partially Reversed by Methionine Restriction Applied in Old Age
Source: Int J Mol Sci. 2021 Nov 20;22(22):12517. doi: 10.3390/ijms222212517 (PMC8623997; doi:10.3390/ijms222212517)
Supplement: Supplementary file 1 [file ijms-22-12517-s001.zip › ijms-1450261-supplementary.pdf]

Article

Age-Related Changes in Lipidome of Rat Frontal Cortex and Cerebellum Are Partially Reversed by Methionine Restriction Applied in Old Age

Mariona Jové [1], Rosanna Cabré [1], Natàlia Mota-Martorell [1], Meritxell Martin-Garí [1], Èlia Obis [1], Paula Ramos [1], Iván Canales [1], José Daniel Galo-Licona [1], Joaquim Sol[1,2,3], Lara Nogueras [1], Pascual Torres [1], Manuel Portero-Otín [1], Victòria Ayala [1], Isidro Ferrer [4,5], Reinald Pamplona [1,*]

1. Supplementary Tables

Supplementary Table S1. List of internal lipid standards used for lipidomic analysis.

Compound	RT in sample	Reference
1,3(d5)-dihexadecanoyl-glycerol	8.2	110537, Avanti Polar Lipids
1,3(d5)-dihexadecanoyl-2-octadecanoyl-glycerol	10.4	110543, Avanti Polar Lipids
1-hexadecanoyl(d31)-2-(9Z-octadecenoyl)-sn-glycero-3-phosphate	7.7	110920, Avanti Polar Lipids
1-hexadecanoyl(d31)-2-(9Z-octadecenoyl)-sn-glycero-3-phosphocholine	7.7	110918, Avanti Polar Lipids
1-hexadecanoyl(d31)-2-(9Z-octadecenoyl)-sn-glycero-3-phosphoethanolamine	7.2	110921, Avanti Polar Lipids
1-hexadecanoyl-2-(9Z-octadecenoyl)-sn-glycero-3-phospho-(1′-rac-glycerol-1′,1′,2′,3′,3′-d5)	7.9	110899, Avanti Polar Lipids
1-hexadecanoyl(d31)-2-(9Z-octadecenoyl)-sn-glycero-3-phospho-myo-inositol	8.1	110923, Avanti Polar Lipids
1-hexadecanoyl(d31)-2-(9Z-octadecenoyl)-sn-glycero-3-[phospho-L-serine]	8.3	110922, Avanti Polar Lipids
26:0-d4 Lyso PC	0.9	860389, Avanti Polar Lipids
18:1 Chol (D7) ester	10.7	111015, Avanti Polar Lipids
D-erythro-sphingosine-d7	2.9	860657, Avanti Polar Lipids
D-erythro-sphingosine-d7-1-phosphate	4.8	860659, Avanti Polar Lipids
N-palmitoyl-d31-D-erythro-sphingosine	7.6	868516, Avanti Polar Lipids
N-palmitoyl-d31-D-erythro-sphingosylphosphorylcholine	6.7	868584, Avanti Polar Lipids
Octadecanoic acid-2,2-d2	4.1	19905-58-9, Sigma Aldrich

RT: retention time

2. Supplementary Figures

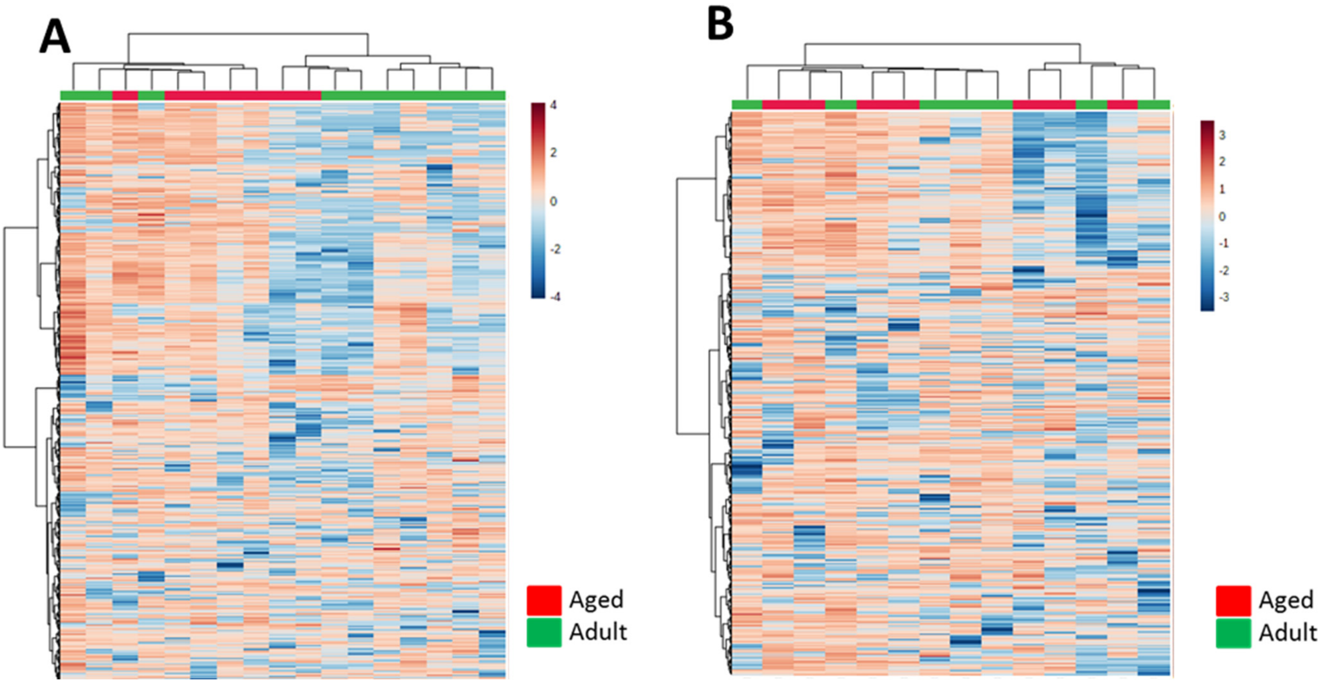

Supplementary Figure S1. Heatmap of hierarchical clustering using all lipid species detected in cerebellum (A) and frontal cortex (B). Each colored cell on the map corresponds to a relative concentration value, with samples in columns and compounds in rows.

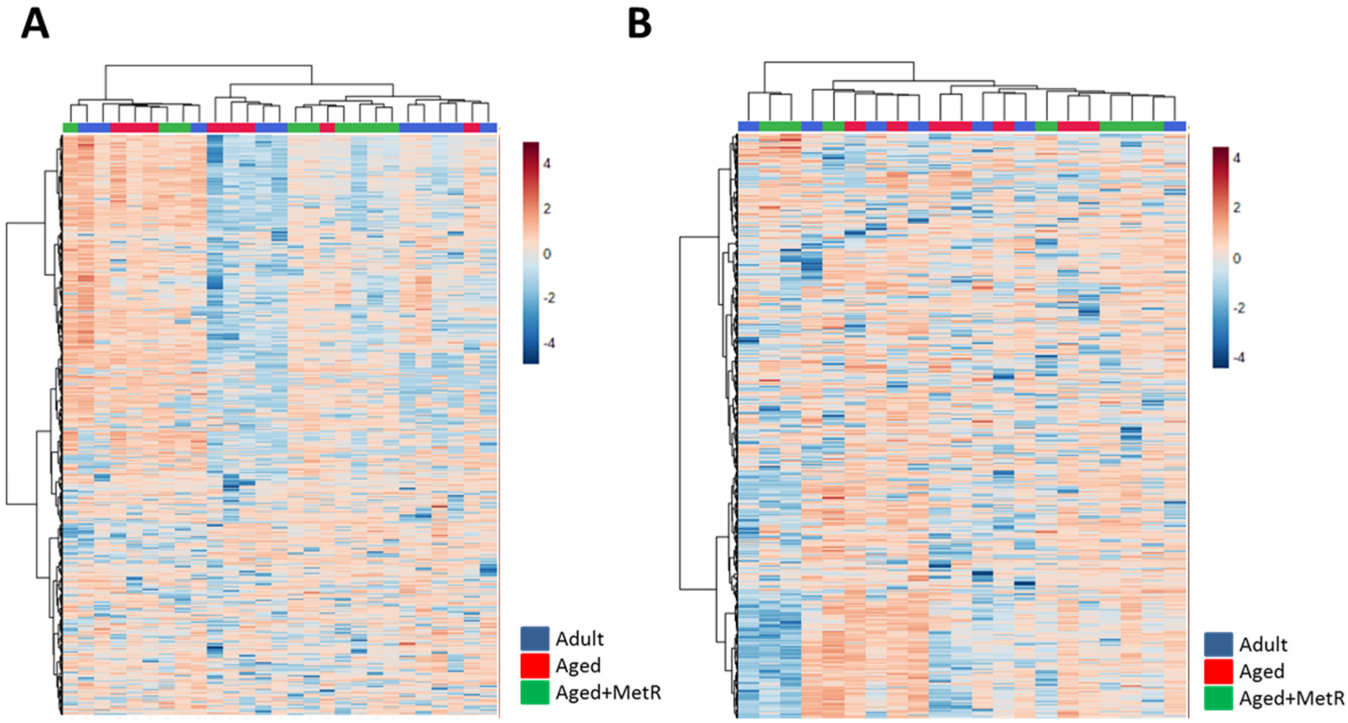

Supplementary Figure S2. Heatmap of hierarchical clustering using all lipid species detected in cerebellum (A) and frontal cortex (B). Each colored cell on the map corresponds to a relative concentration value, with samples in columns and compounds in rows.

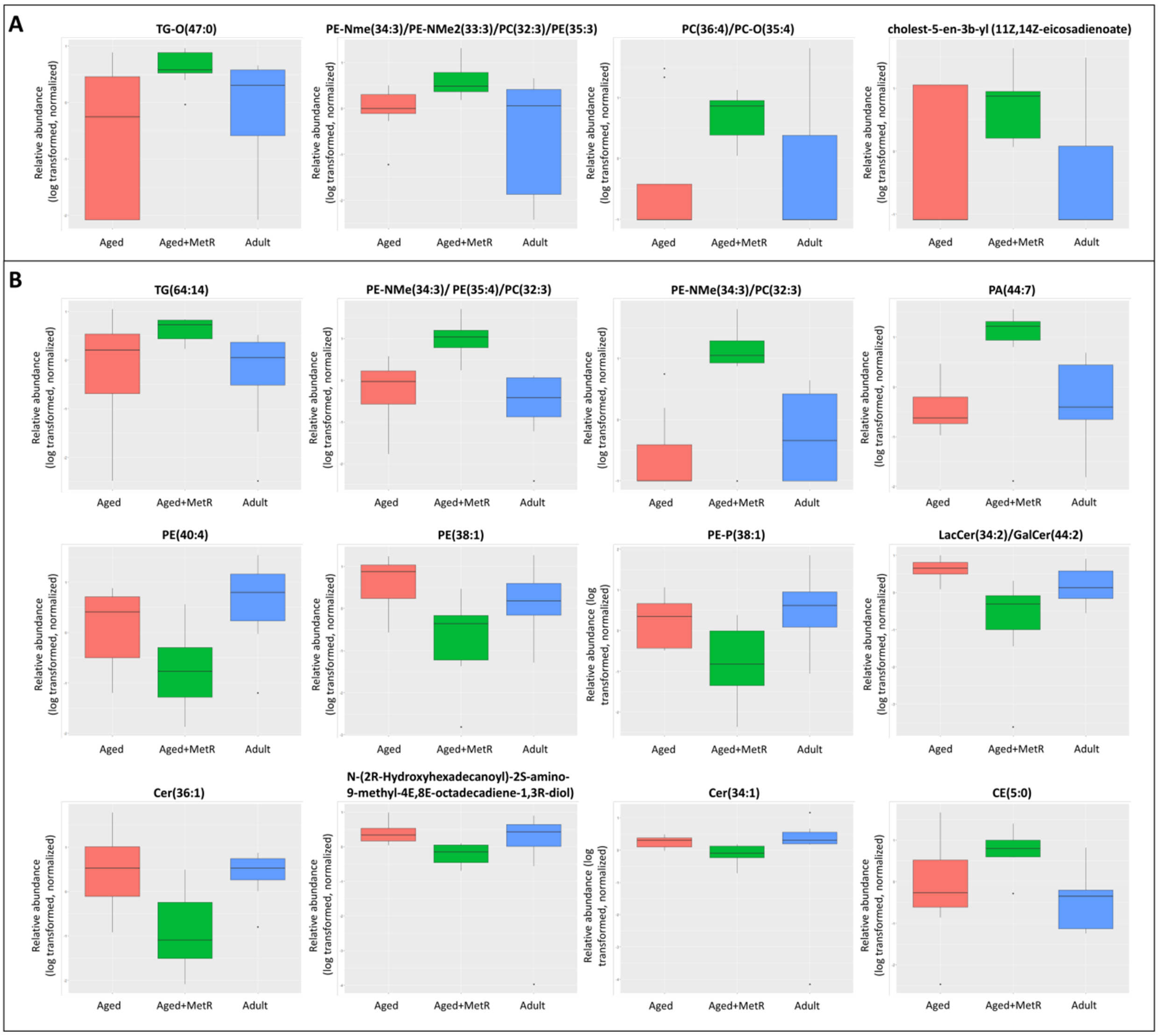

Supplementary Figure S3. Box and Whiskers plot of cerebellum (A) and frontal cortex (B) biomarkers of MetR diet.

3. Supplementary Materials and Methods

Untargeted lipidomic analysis: Lipid extraction

In order to precipitate tissue protein fraction, 5 µl of water and 20 µl of methanol were added to 10 µl of tissue homogenate sample and samples were vigorously shaken for 2 min. Then 250 µl of methyl tert-butyl ether (MTBE) containing internal lipid standards were added and samples were immersed in a water bath (ATU Ultrasonidos, Valencia, Spain) with an ultrasound frequency and power of 40 kHz and 100 W respectively, at 10 °C for 30 min. Finally, 75 µL of water were added to the mixture and organic phase was separated by centrifugation at 1400 g at 10 °C for 10 min. Lipid extracts contained in the upper phase were collected and subjected to LC-MS analysis. The list of internal lipid standards used and added to samples is indicated in Table 9. All lipid extracts were pooled and used as quality controls (QC).

Untargeted lipidomic analysis: Identity assignation.

In order to identify the lipid species, the compounds of interest were fragmented in the same equipment under the same conditions used for the initial analysis, using as precursor ions the m/z obtained by MS1 as well as its specific retention times for each compound. Different voltages as collision energy (10, 20 and 40 V) were used to obtain the spectra of product ions in MS2 mode for each compound.

Using data obtained both with LC, MS1 and MS2 was used for identification, using three different approaches:

As a first approach, the compounds were searched using the parent and product ions of different voltages in the HMDB database. In case that some fragment would be an ion compatible with the corresponding lipid class, which coincided to the parent ion putative annotation and its retention time, the lipid was assigned to that class. In case of not having any class information compatible fragment, as well as to be able to identify the compound at a more specific level, the spectra with a higher score were selected and filtered visually taking into account that a) 2 or more ions matched between the experimental spectrum and that of the database, b) that the relative intensities were similar in the two cases, and c) that conditions a) and b) should be positive by two different collision energies.

The second approach used LipidMatch. The parent of each compound and its product ions obtained after compound fragmentation are searched into LipidMatch's own database. LipidMatch assigns each feature to a lipid match based on exact mass (MS1 level), then it matches in-silico fragmentation for lipids to the fragments found in experimental spectra (MS2 level). For each fragment match, it filters those meeting the minimum intensity criteria and the minimum MS2 scans (in our case set on 100 and 1). Then it filters again the list using the necessary fragments for confirmation, which vary among lipid classes (for example PC(16:0/22.6) needs R1COO- and R2COO-). And finally, it gives the results based on lipid structural resolution; 1) by fatty acids and class (MS2 level) 2) by class (MS2 level) or 3) by precursor mass (MS1 level). When both the parent ion and the fragmented ions match the program gives its potential identities ranked by summed fragment intensity. In case of discrepancies when more than one possible class where possible, the ones with the most intensity were chosen.

Finally, the third approach used MS-DIAL and fragments were compared with those included in MS-DIAL internal database. Data files were first converted to Abf format using Abf Converter. MS1 tolerance was set at 0.01Da and MS2 tolerance at 0.1Da. Minimum peak height was set at 100 amplitude. Adducts M+H, M+H-H2O, M+NH4, M+Na, M+K were taken into account for positive ionization and M-H, M-H2O-H, M+CH3COO for negative ionization. The rest of items were kept at default. The criteria used for identity confirmation were the same used in HMDB search.

Once assigned the different classes or identities for the different compounds, we performed a manual check of each, comparing the LipidMatch results with the HMDB results and the MS-DIAL results. If there were discrepancies, we checked the experimental spectrum to see which was more similar as described above for HMDB search.

In the event that the spectra obtained from the same compound using different collision energies did not match with those described in databases, the potential identity was left as unknown as it had not been confirmed by MS2. If there was no spectrum to compare with but the compound coincides in mass and retention time with that lipid class, the result obtained by MS1 was used.

Fatty acid profile

Fatty acids of total lipids from rat cerebellum and frontal cortex homogenate were analyzed as fatty acid methyl esters (FAME). From samples containing 0.5 mg of proteins, total lipids were extracted and incubated in 2 ml of 5% methanolic HCL at 75 °C for 90 min. FAMEs were extracted by adding 1 ml of saturated NaCl solution and 2 ml of n-pentane and centrifuged at 4400 rpm a 4 °C for 15 min after mixing. The phases with n-pentane were separated and evaporated under N_2 gas and finally dissolved in 80 µl of carbon disulfide.
